# Content analysis of suicidal notes - the verbal behaviour

**DOI:** 10.1192/j.eurpsy.2024.1626

**Published:** 2024-08-27

**Authors:** J. D. Fekete, S. Fekete

**Affiliations:** ^1^Dept of Language and Communication; ^2^Dept of Psychiatry, University of Pecs, Pecs, Hungary

## Abstract

**Introduction:**

There have been numerous studies on attempted and completed suicides in an attempt to understand better the phenomenon - the tragedy- of self-destructive behaviour through the analysis of the suicidal notes – their last personal documents, that many individuals write before carrying out suicide. Understanding and interpretation of these analyses could happen on several theoretical frameworks and background Research has systematically demonstrated what most clinicians assume, namely that individuals sharing significant patterns of nonverbal behavior express these tendencies in their manner of speaking and writing (e.g. in suicidal notes, farewell letters) . Through this research – by analyzing these texts, documents,” messages”,- was it possible to study not only semantic,and lingistic aspects of them but likely also as manifestations of psychological defense or coping mechanisms or reflected psychopathology in speech by isolating categories. A number of clinical investigations have been carried out to associate speech pattern and verbal style (spoken or written) with these psychopathological states

**Objectives:**

The purpose of the present study is to understand better of written “suicidal” communication; to analyze suicidal notes - namely, as last “messages” of the self destructed individuals in suicidal notes in an empirical sample.

**Methods:**

The present study compared the content of suicide notes (n=113) from attempted suicides, completed suicides and a non-suicidal controls. The content analysis examined formal, syntactical characteristics, as well as speech patterns and verbal expressions (Weintraub method, Absolutists index, SPSS, - Anova, KW)

**Results:**

The notes from completed suicides had significantly higher scores for heteroaggression (blaming others, evaluators) negations, absolutistic expressions, nonpersonal references and lower scores for expression of feelings. Sex (male versus female) and age had no impact on these differences.

**Conclusions:**

The suicide notes had reflected irrational thinking, characterized by frequent negation, and absolutistic words, self-preoccupation, high scores for emotional categories and a tendency toward polarized thinking These results may help in the understanding of the psychodynamic background or suicidal individuals’ risk assessment, in clinical work or in suicide hotlines, but also in prevention

**Disclosure of Interest:**

None Declared

